# Assistance dogs provide a useful behavioral model to enrich communicative skills of assistance robots

**DOI:** 10.3389/fpsyg.2013.00971

**Published:** 2013-12-24

**Authors:** Márta Gácsi, Sára Szakadát, Ádám Miklósi

**Affiliations:** ^1^MTA-ELTE Comparative Ethology Research GroupBudapest, Hungary; ^2^Department of Ethology, Eötvös Loránd UniversityBudapest, Hungary

**Keywords:** assistance robots, dog–human cooperation, dog behavioral model, displacement behaviors, problem solving strategy

## Abstract

These studies are part of a project aiming to reveal relevant aspects of human–dog interactions, which could serve as a model to design successful human-robot interactions. Presently there are no successfully commercialized assistance robots, however, assistance dogs work efficiently as partners for persons with disabilities. In Study 1, we analyzed the cooperation of 32 assistance dog–owner dyads performing a carrying task. We revealed typical behavior sequences and also differences depending on the dyads' experiences and on whether the owner was a wheelchair user. In Study 2, we investigated dogs' responses to unforeseen difficulties during a retrieving task in two contexts. Dogs displayed specific communicative and displacement behaviors, and a strong commitment to execute the insoluble task. Questionnaire data from Study 3 confirmed that these behaviors could successfully attenuate owners' disappointment. Although owners anticipated the technical competence of future assistance robots to be moderate/high, they could not imagine robots as emotional companions, which negatively affected their acceptance ratings of future robotic assistants. We propose that assistance dogs' cooperative behaviors and problem solving strategies should inspire the development of the relevant functions and social behaviors of assistance robots with limited manual and verbal skills.

## Introduction

In order to enhance the effectiveness and quality of human-robot interactions, users should be able to work with assistance robotic systems in a natural way. Presently, most assistance robots try to mimic human capacities, embodiments, and behaviors, even though neither their function nor their abilities make them comparable to a human assistant. Here, we argue that the adaptation of some relevant behavior patterns observed in assistance dog–owner interactions could improve the ability of assistance robots of different embodiments to provide successful social and physical assistance to people with disabilities. In order to design such robotic assistants, we need to examine owner–dog interactions with a special focus on the situations that the assistant robot would face while performing its duty.

The presented experiments are part of our broader project that aims to reveal relevant aspects of human–dog interactions, which could serve as a model to design successful human–robot interactions. Through applying an ethological approach to develop believable and efficient social interactions for social robotics, our studies so far have ranged from finding the plausible channels of communication in human–robot–dog swarms applied for specific rescue purposes (Gerencsér et al., 2013), through adapting the behavioral characteristics of dog–human attachment to robot-human dyads (Kovács et al., 2011), to modeling exploratory behavior of assistance robots designed for home-care applications (Numakunai et al., 2012).

Considering the growing geriatric population of western industrialized societies, it will be an absolute necessity in the near future that the elderly and disabled are able to communicate and cooperate successfully with home assistance robots. From the several definitions of robots that assist people (e.g., Feil-Seifer and Matari, 2005), here we refer to assistance robots as autonomous mobile robots that, in order to provide assistance to people with disabilities, are able to cooperate with humans and have the potential to manipulate the environment while performing a wide array of tasks (Chen et al., 2013). There are many different endeavors to design technological aids that help the rehabilitation of the physically disabled, ranging from intelligent wheelchairs to different types of assistance robots (Amat, 1998). Most of these robots are designed for specific roles and functions. Assistance systems (consisting of more than one robot) are also developed (Yamazaki et al., 2011). For example, the small home robot, Mamoru-Kun, is able to inform its owner where an object is located by communicating verbally or by pointing at the object. It can also cooperate with his humanoid buddy and ask it to get the object for the user. This bigger robot is able to clean up rooms, manipulate dishes, open, and close doors, do the laundry, and even learn from its mistakes.

In spite of the extensive and wide ranging research, the commercialization of autonomous assistance robots is not planned for another 10 years. One of the main difficulties is designing robots that people do not find disturbing, can easily operate and communicate with, and that they can accept as social partners (Dautenhahn, 2007). To make humans feel that their interactions with the robot are natural, it is essential that in addition to providing technical aid, the robot must also act in accordance with the given social context and shows relevant social abilities (Bartneck et al., 2004).

Based on the general assumption that people find relatable companions easier to deal with, the most common strategy, both in scientific and commercial environments, is to design humanoid robots that have (seemingly) humanlike features and capabilities. One of the potential problems with the use of anthropomorphic behaviors lies in the controversy that humanoid robots are likely to raise the user's expectations in terms of the robot's capabilities and interactional affordances, but present-day robots are not able to fulfil these expectations (Miklósi and Gácsi, 2012). Recently, an alternative suggestion has come up arguing for the use of non-human social animals as models for robot social behavior, emphasizing the fact that human–animal interaction provides a rich source of knowledge for designing social robots that are able to interact with humans under a wide range of conditions (Dautenhahn, 2004). The idea that, in addition to its technical help, a robotic assistant could be a suitable companion, directed the attention of researchers to use pets as potential behavioral models (Jones et al., 2008).

The function and relatively limited abilities (compared to that of a human assistant) of assistance robots suggest an asymmetrical social relationship between the human user and the robot, which is in many ways similar to the dog–owner relationship (Topál et al., 2005). Assistant dogs can successfully communicate and cooperate even with a disabled owner who cannot speak clearly, and they show social behaviors, such as attachment, which humans can easily understand without extensive prior learning. This way, beside technical assistance, these trained dogs provide social support for their owners (Fallani et al., 2006).

It has already been suggested that using assistance dog models to design subtle motor behaviors for the manipulation skills of assistance robots is a highly beneficial and cost effective strategy (Nguyen and Kemp, 2008). We would like to go even further and give dogs a more ambitious role: we suggest that the social behavior of such robots should be developed on the basis of assistance dog–owner interactions. Some robots have already been proposed for therapy purposes, for example Sony's robot pet, AIBO, was found to stimulate social interaction among nursing home residents with dementia (Kramer et al., 2009). It is important to stress however, that our aim is not to develop a doggy robot like AIBO, because we do not believe that a robot that physically resembles a dog would be an ideal assistant in more complex therapy situations, or for helping people with disabilities. Rather, we propose the use of an embodiment that is optimally fitted for the specific function of the given robot (without the limitations of dog anatomy), and the application of specific behavioral models for different technical and social functions, for example, assistance dogs for persons with disabilities. In order to do so, we investigate a number of rich, relevant, and realistic contexts, in which we can observe and evaluate the joint activities of dogs and humans. Our aim is to identify simple, basic behaviors available to even a mechanical-looking embodiment, such as orientation, proxemic behavior, gross body movement, and the comprehension of gestural and verbal communication from a human partner. After extracting the relevant set of behavior elements based on the desired function, we can adapt the applicable ones to robots of different embodiments and capacities. Our first study supporting the dog-model idea applied one of the typical scenarios between service dogs and their disabled owners: assisting in object transportation (Syrdal et al., 2010). In this study, an appearance-constrained Pioneer robot used dog-inspired affective cues to communicate with its owner and a guest in a fetch and carry scenario. The findings suggested that even limited modalities of non-verbal expression offered by the robot (proxemics, body movement and orientation, camera orientation) were effective for developing/helping the communication. In the next application of our idea, the dynamics of feedback processes during the interaction between a robot used as a hearing aid and human subjects who played the role of the deaf owner were investigated (Koay et al., 2013). The behavior patterns of the robot, which had no arms or verbal capacities, were designed based on the interactions between hearing dogs and their deaf owners. Dog–inspired behavior sequences and decision making strategies were used to program and control the robot during the trials. Findings indicated that untrained and uninformed participants could correctly interpret the robot's actions, and that head movements and gaze directions during signaling, leading, and feed-back processes were important and effective for communicating the robot's intentions.

In the present paper, we had three aims. First, we wanted to provide deeper insight into one typical interaction by detailed analyses of the behavior of a large and diverse sample during a carrying task. Second, to develop a life-like and flexible behavioral set for assistance robots it is inevitable to prepare them to handle problem situations. These robots will certainly face several insoluble tasks and could not always meet their users' requirements. We suggest studying assistance dogs' behaviors in such situations to provide a useful guide to developing robot behavior. Finally, we wanted to assess assistance dog owners' views about their dogs' relevant behaviors and compare their attitudes toward an assistant dog and an imaginary assistant robot.

In Study 1, we investigated the typical behaviors and interactions that can be observed in dog–owner dyads when they perform a carrying task. We examined whether there were any differences in their behaviors depending on how experienced the dyad was and on whether the owner was a wheelchair user or not. Wheelchair use presents a specific limitation for disabled persons, as it may require specific interaction types or capacities from an assistant (dog or robot).

In Study 2, we observed the behavior of a subsample of dogs facing two different types of insoluble tasks commanded by the uninformed owners. Preliminary observations have shown that dogs in problem situations display (i) communicative behaviors to signal the problem to the owner (vocalization, gazing/approaching the owner etc., e.g., Miklósi et al., 2003), (ii) continue engagement with the task or try to perform some alternative activities that are connected to the original task to some extent and/or (iii) tend to show displacement activity. Displacement behaviors emerge both in humans (Barrett, 2005) and non-human animals (Maestripieri et al., 1992) in conflicting situations. For instance, when dogs find themselves in a situation where they are unable to solve a task or a problem, they show typical out-of-context behaviors (e.g., mouth-licking, yawning, sniffing the ground, scratching the ground, pacing up and down between the owner and the designated location), which reflect their confusion (Beerda et al., 2000). Exhibiting such behaviors should not necessarily be regarded as intentional communication of the situation's ambiguity, and might only be effective if the owner is sensitive or experienced enough to read these behavioral signals. However, a major approach of social psychology (Miller and Leary, 1992; Keltner, 1995) sees the roots of similar human behaviors in the appeasement behaviors of animals. These unintentional actions have appeasement-related functions, as they are able to pacify partners in case of social transgressions by reducing aggression and evoking social approach, thus restoring the social interaction and relation.

We expected that the above three types of behaviors of assistance dogs can influence or inhibit the negative feelings of the owners, similarly to “guilty” behavior of pet dogs after transgression (Hecht et al., 2012). Therefore, in Study 3, we used a questionnaire to assess the typical reactions of assistance dog owners to the situations modeled in Study 2. In general, when machines cannot fulfill their requirements, people are disappointed and annoyed. Preliminary observations showed however, that when a trained assistant dog could not execute a command, owners were more indulgent and forgiving. Our questionnaire aimed to investigate whether behaviors like those displayed by the dogs in Study 2 have a positive influence on owners' reactions. Moreover, based on the questionnaire data, we could compare owners' attitude toward their dog and toward a potential future robotic assistant.

## Study 1—carrying

In this study, the dog was supposed to carry a basket from a start point to a target place, which is a cornerstone of their training. This task may include picking up, carrying and placing objects at home or outside the house. While having a dog assist in these activities increases the disabled owner's independence, it still requires some actions from the individual. An assistant capable of properly performing the task has to be able to (i) get ready for performing a task when indicated, (ii) understand human communicative cues to identify the target object, (iii) hold and carry the basket, (iv) follow, escort, or go ahead of the owner (with continuous feedback from the owner's orientation/instructions), (v) put down the basket on command at the target point. As we wanted to reveal the dyad's spontaneous cues and behaviors during the interactions, the only instruction for the owners was that they must not touch the dog or the basket during the task (however they could go with the dog).

### Methods

We confirm that informed consent was obtained from all subjects, and the experiment was performed in accordance with relevant institutional and national guidelines and regulations (Eötvös University, University Institutional Animal Care and Use Committee).

#### Subjects

We observed 32 dog–owner dyads. All dogs were trained assistance dogs; certified therapy dogs or service dogs for the disabled. All dogs had been trained by the Dogs for Humans Foundation, Hungary and they were tested with their owner (O). There were 11 males and 21 females among the Os. Half of the dyads were novice (having worked together for less than a year), with dogs having the same training as the other group, but not as much experience in various contexts. The other 16 dyads were experienced; these dogs had been working with their Os for multiple years. In both the novice and experienced groups, 8 Os were wheelchair users while the other 8 Os were able to walk. All dogs were more than 1 year old and they were of different breeds (*Novice*: 4 males, 4 females, mean age: 4.0 years (range: 1–7 years), breeds: Belgian sheepdog, sheltie, golden retriever, border collie, Airedale, 2 Labrador mixes; *Novice-wheelchair*: 5 males, 3 females, mean age: 2.6 years (range: 1–7 years), breeds: Belgian sheepdog, golden retriever, cavalier, cocker, poodle, Labrador mix, Leonberger mix, mongrel; *Experienced*: 3 males, 5 females, mean age: 3.8 years (range: 1.5–7 years), breeds: 3 Belgian sheepdogs, vizsla, Labrador mix, 3 mongrels; *Experienced-wheelchair*: 5 males, 3 females, mean age: 5.6 years (range: 2.5–9 years), breeds: 3 Belgian sheepdogs, 3 golden retrievers, collie mix, Labrador mix). The training of these dogs was based on the principle that they should be eager to please their O, that is, they must do their best to find out what task the owner is trying to communicate and to cooperate in the task's execution, even if the human's communication is not completely clear.

#### Procedure

**Figure 1:**
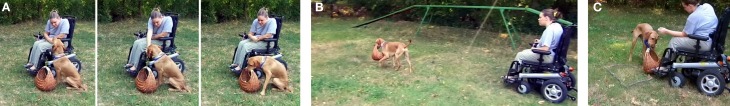
**Snapshots from the video on the 3 phases of the test: (A)** picking up, **(B)** carrying, and **(C)** placing the basket.

The tests were conducted in a park that was familiar to the dogs. The dog and O were positioned at the start point. The experimenter (E) placed the basket in front of them. The target place was positioned 10 m from the start point and marked by three 80 cm long sticks forming an equilateral triangle (never used as a target place before). The behavior of the dyad was video recorded from the side by E from a distance of 5–6 m. The records were analyzed later. (Figure 1)

#### Data analysis

During the analysis, our main focus was on the communication between the dog and O (paying and getting attention; communicating target object, target location and expected actions) and the dynamics of their movements (durations, relative position of the dog while carrying the object). The following behavior variables were coded: joint attention (O and dog gaze at each other) (yes-no), number of verbal instructions, O's pointing gestures (yes-no), relative duration of going ahead–beside–behind relative to O when performing the carrying task (time percentage), duration of the picking up (s) and placing (s) tasks. (The duration of the carrying phase was not analyzed because wheelchair users' speed was limited on the rough surface.) Multivariate analysis of variance was applied to compare durations (picking up, placing) and number of verbal instructions in the four groups (using experience and wheelchair use as factors). As the data of relative duration of the dogs' position were not normally distributed, Kruskal-Wallis test and Mann-Whitney test were used to compare the different groups. Benjamini correction was applied to control for multiple comparisons. The owners' verbal instructions could be categorized as commands (verbs), name of dog, name of object (“basket”), praises (e.g., “well done”), and inhibitions (e.g., “no”, “don't”) in all three parts of the test. Inter-observer agreements for all variables were assessed by means of parallel coding of 8 dogs' tests (2 dogs were randomly chosen from each group). High values for agreement between the two observers were found in all cases (Kappa coefficients are: 1 for joint attention, 0.84 for duration of position relative to O, 0.78 for number of verbal instructions, 0.91 for picking up, and 0.81 for placing).

### Results

#### Types of verbal communications

The proportion of the categories during the entire test duration was as follows: commands (verbs) 63%, name of dog 14%, name of object 8%, praises 13%, and inhibition 2%.

#### Phase I: picking up

We observed joint attention in all dog–owner dyads at the very beginning of the task. In most cases, eye-contact was spontaneous (*N* = 22), that is, the dog oriented to O right after having been seated at the start point. If the dog oriented somewhere else (*N* = 10), O tried to attract its attention by calling its name before giving any instructions. After this, all but four Os pointed at the basket when communicating the task to the dog. Three of the exceptions were from the experienced/wheelchair user group, who either needed both hands for driving the mechanical wheelchair, or could not move their arms. All four Os however, “pointed” toward the basket with their head. (Table 1) The number of verbal instructions and the duration of the task showed significant positive correlation (*r* = 0.645, *p* < 0.001), and seemed to vary according to the experience; novice dogs needed more verbal instructions [*F*_(1, 28)_ = 6.563, *p* = 0.016] to execute the task [the effect on duration did not remain significant due to Benjamini correction; *F*_(1, 28)_ = 4.457, *p* = 0.044]. Wheelchair use did not affect these behaviors [verbal instructions: *F*_(1, 28)_ = 0.632, *p* = 0.433; duration: *F*_(1, 28)_ = 0.485, *p* = 0.487].

**Table 1:** Number of dyads where joint attention and/or pointing gesture could be observed during the three phases of the task (^*^marks pointing with head).

	**Picking up**		**Carrying**		**Placing**	
	**Joint attention**	**Point**	**Joint attention**	**Point**	**Joint attention**	**Point**
Novice	8	7 (+1^*^)	1	0	4	2
Novice Wheelchair user	8	8	7	4	7	7
Experienced	8	8	4	3	8	7
Experienced Wheelchair user	8	5 (+3^*^)	6	2	4	5

#### Phase 2: carrying

**Figure 2:**
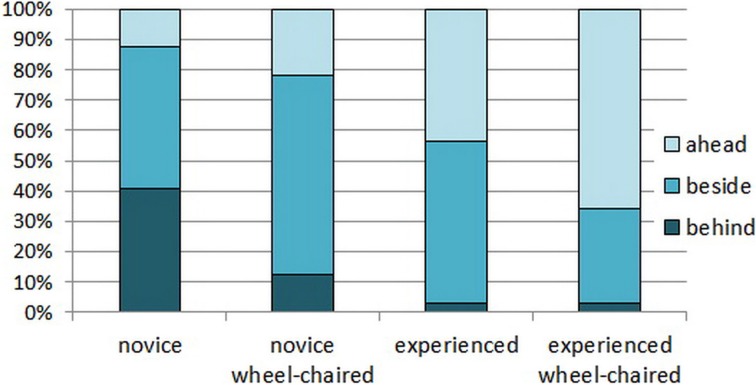
**Relative durations of carrying the basket in the three possible positions relative to the owner in the four groups**.

Joint attention and pointing gestures were also observed in this phase but they were not typical (see Table 1). Neither experience [F_(1, 28)_ = 4.982, *p* = 0.034, due to Benjamini correction], nor wheelchair usage affected the number of verbal instructions [*F*_(1, 28)_ = 2.184, *p* = 0.151]. The position of the dogs during the basket carrying showed an interesting pattern. Compared to the novice group, experienced dogs tended to carry the basket longer by going ahead of O (*Z* = −2.596, *p* = 0.009) and shorter by following the owner (*Z* = −2.654, *p* = 0.008) compared to novice dogs that typically carried the basket beside or behind the owner. There was no difference in the relative duration of escorting O (*Z* = −1.04, *p* = 0.298). Using a wheelchair in itself did not make a general difference in this respect (ahead: *Z* = −1.031, *p* = 0.303; beside: *Z* = −0.096, *p* = 0.923; follow: *Z* = −0.697, *p* = 0.486), and the result of post hoc tests that dogs had the tendency to carry the basket going ahead especially in the experienced wheelchair user group did not remain significant after applying Benjamini correction [*X*^2^_(3)_ = 8.09, *p* = 0.044]. (Figure 2)

#### Phase 3: placing

Joint attention and pointing gestures were used frequently but not by all dyads (Table 1). The number of verbal instructions and the duration of the task strongly correlated (*r* = 0.95, *p* < 0.001), and in case of both variables we found significant main effect of experience (verbal instructions: *F*_(1, 28)_ = 8.119, *p* = 0.008; duration: *F*_(1, 28)_ = 10.382, *p* = 0.003) and wheelchair use [verbal instructions: *F*_(1, 28)_ = 8.89, *p* = 0.006; duration: *F*_(1, 28)_ = 12.396, *p* = 0.001]. The significant interactions of experience and wheelchair use (verbal instructions: *F*_(1, 28)_ = 8.688, *p* = 0.006; duration: *F*_(1, 28)_ = 8.739, *p* = 0.006) revealed that especially the novice wheelchair user group needed more time and instructions to successfully place the basket.

## Study 2—inhibited tasks

This study was planned primarily to activate, observe, and describe the behavior of dogs in two typical conflict situations, not to test specific hypotheses. We presented the dogs with an insoluble version of a simple retrieving task in two test situations. Based on the results of earlier studies, we assumed there would be three types of responses: trying hard to perform the/some task, communication with the owner, and displacement behaviors.

### Methods

We confirm that informed consent was obtained from all subjects, and the experiment was performed in accordance with relevant institutional and national guidelines and regulations (Eötvös University, University Institutional Animal Care and Use Committee).

#### Subjects

A representative subsample of Study 1 (based on availability); 19 assistance dog–owner dyads were tested. (Novice group: *N* = 4, novice wheelchair user group: *N* = 4, experienced group: *N* = 5, experienced wheelchair user group: *N* = 6.)

#### Procedure

The two tests took place in the same room (5 × 3 m) at the Department of Ethology, Eötvös University, Budapest, which was unfamiliar to the dogs. The dogs entered the room together with O and were allowed to explore for a few minutes before the first test.

Both tests were simple fetch and carry tasks and always followed each other in a fixed order with a 10 min break. In Test 1, the task was insoluble because in the test trial the basket to be carried was not at the place where it was supposed to be. In Test 2, dogs encountered a non-cooperative experimenter (E) who inhibited the fulfillment of the task by not handing over the basket. In both tests the same trained female E manipulated the basket. The tests were video recorded from 4 different angles (using cameras fixed to the walls) and analyzed later.

### Test 1

There were two chairs in the room, one in the middle for O (if O did not use a wheel chair) and one in the corner for the dog's leash. As a starting position, the dog sat next to its O off leash. In the other end of the room there was a small cupboard and a barrier attached to it making one of the corners invisible to the dog and the owner. Opposite of the barrier there was an open door.

**Figure 3:**
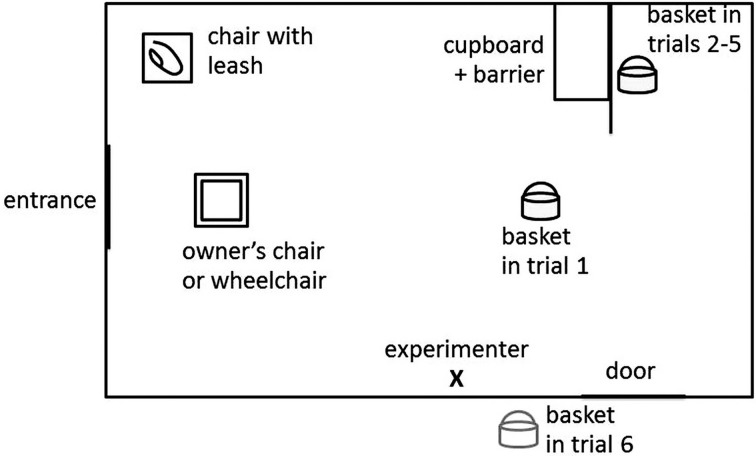
**The schematic drawing of test situation in Study 2/Test 1, showing the position of the participants and the place of the basket in different trials**.

Prior to the test, E explained the scenario to O without mentioning the final inhibited trial. Therefore, neither the dog nor the owner knew that in the last trial the basket would not be at its place. E laid down two rules: (1) O is allowed to use only the following 3 words: “bring,” “basket” and “come” (to avoid the use of instructions such as “seek” or “find”) and (2) after first sending the dog to fetch the basket, O is allowed to talk to the dog only if the dog gazes at her/him. (Figure 3)

#### Warm up phase (5 trials)

The test began with basic basket retrieving tasks. First, E showed the basket to the dog, put it down on the floor in the middle of the room about 2 m from the dyad, went to the wall and stood still. O sent the dog to retrieve the basket, and praised the dog when it executed the task. After this, the dog returned to its original position at O's side. In the two following trials, after showing the basket to the dog, E hid the basket behind the barrier, and the dog had to retrieve it from there. E stood at the wall when O sent the dog for the basket. In the next two trials a new element was introduced in order to prepare the dog for the test phase. O had to cover the dog's eyes and also close his/her own eyes before E hid the basket behind the barrier. Then E slowly closed the door (just to get the dog and O used to the noise), stepped back to her place at the wall, and instructed the O to open her/his eyes, uncover the dog's eyes and execute the retrieving task in the same way as before.

#### Testing phase

The sixth trial was the inhibited one. It was similar to the last two warm up trials, except that when the eyes of the dog and O were closed, right after placing the basket behind the barrier (to mimic the usual noises), E quietly removed the basket to the neighboring room (far enough from the door, so that the dog was not able to smell it from the experimental room), closed the door, returned to her usual position and instructed the owner to execute the same procedure as usual. The duration of the trial was 2 min, measured by E from the moment the dog faced with the lack of the basket behind the barrier. E stood still at her usual place watching the dog. After 2 min, E opened the door and gave the basket to the dog, who returned it to O and was praised.

#### Data analysis

The following variables were coded during the 2 min of the test phase: latency of looking at O (s) and E (s), duration of looking for basket in 1 m area around cupboard (s) and at other places in the room (s), and occurrence of approaching owner (yes-no), vocalization (yes-no), displacement behavior (any of the following): yawning, stretching, paddling, scratching itself, licking its lips, shaking (yes-no), fetching/manipulating other object (yes-no). Inter-observer agreements for all variables were assessed by means of parallel coding of 4 randomly chosen dogs' tests. High values for agreement between the two observers were found (Kappa coefficients are: 0.86 looking at O, 0.83 for looking at E, 0.81 and 0.78 for looking for basket at cupboard/other places), Coders' evaluation fully agreed on all occurrence data.

#### Result

**Figure 4:**
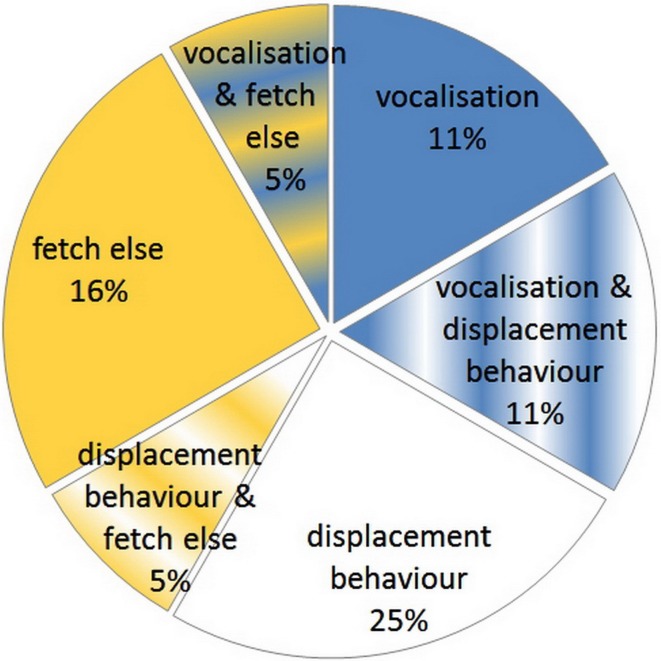
**Proportion of dogs that vocalized, exhibited displacement behaviors, fetched other objects or exhibited some combination of these behaviors when they could not find the object the owner asked for**.

After visiting the basket's usual place behind the barrier, dogs kept looking around the cupboard for an average of 35 s, and looked throughout the rest of the empty room for an average of 58 s. (**Video**) All but one of the dogs looked at O at least once during the inhibited trial; the mean number of gazes was 4.2, and the mean latency of the first gazing was 32 s. A large proportion of dogs (63%) also approached O, and 74% of them even gazed at E. Out of the 13 dogs that looked at both persons 11 looked first at O. Vocalization was not typical, but 27% of the dogs vocalized while confronting the problem. Some displacement behavior was exhibited by 31% of the dogs at least once, and 26% of the dogs fetched/manipulated some other object, mainly the leash or tiny pieces of the test set up (tape) (Figure 4).

### Test 2

This time there were three chairs in the room, one in the middle for O (for non-wheelchair users), one in the corner for the dog's leash and one for E placed about 2 m from the dyad in the middle of the room. As a starting position, the dog sat next to its O off leash. E showed the basket to the dog, sat down onto her chair, placed the basket on the floor while still holding its handle, and instructed O to command the dog to bring back the basket. However, when the dog got hold of the basket, E did not hand it over, but held on to it. The trial lasted for 1.5 min measured by E from the moment the dog first got hold of the basket. The same rules for giving instructions to the dog were applied as in Test 1. After 1.5 min E released the basket, so the dog could return it to O and be praised.

#### Data analysis

The following variables were coded: latency of looking at O (s), duration of pulling the basket (s), occurrence of looking at E when not manipulating the basket (yes-no), approaching O (yes-no), vocalization (yes-no), displacement behavior (any of the following): yawning, stretching, paddling, licking its lips, shaking (yes-no), fetching/manipulating other object (yes-no). Inter-observer agreements for all variables were assessed by means of parallel coding of 4 dogs' tests (randomly chosen but different ones than in Test 1). High values for agreement between the two observers were found (Kappa coefficients are: 0.82 for looking at O, 0.93 for pulling basket), Coders' evaluation fully agreed on all occurrence data.

#### Result

**Figure 5:**
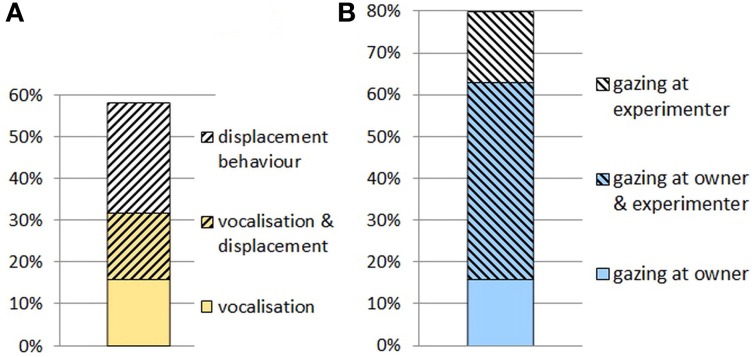
**(A)** Proportion of dogs that vocalized, exhibited displacement behaviors or did both when the experimenter did not hand over the object. **(B)** Proportion of dogs that gazed at the owner, the experimenter or at both of them when the experimenter did not hand over the object.

Most importantly, all dogs tried to take the basket by pulling it; the mean duration for pulling was 28 s. In this test only two dogs fetched another object for O. When E did not allow them to take the basket, 63% of dogs looked at O, the mean latency for gazing was 33 s. Moreover, 32% of the dogs approached O, and 67% of them gazed at E. One-third of the dogs (32%) vocalized (high pitched bark or whine) orienting to the non-cooperative E and/or O. A relatively large proportion of dogs (42%) exhibited at least one form of displacement behavior. (Figure 5)

## Study 3—questionnaire

### Methods

#### Subjects

We asked the assistance dog Os of the Dogs for Humans charity to fill in a short online questionnaire. We received 40 data sets from 8 males and 32 females, whose age distribution was as follows: <18: 5%, 18–30: 25%, 30–50: 57.5%, >50: 12.5%. Out of this group, 29 Os participated in the behavioral tests.

#### Procedure

The ten questions were about Os' (i) relationship with their dog, (ii) reactions in problem situations depending on the dog's responses, (iii) attitudes toward an imaginary robotic assistant (Table 2). The questions could be answered using a five-point scale, scoring how much the subject felt the listed feature/behavior is typical or characteristic (1: not at all—5: extremely) or how much the given behavior of the dog would help O understand and handle the situation (1: not at all—5: entirely).

**Table 2:** **The questions and results in Study 3: The median and quartiles of assistance dog owners' scores (1-5)**.

**No**	**Question**		**Median**	**Lower quartile**	**Upper quartile**
1	To what extent are you satisfied with the work of your dog?		**4.5**	4	5
2	To what extent do you consider your dog an emotional partner?		**5**	4	5
3	How much do you talk to your dog for the following reasons?	I give orders	**4**	4	5
		I ask questions	**4**	3	5
		I talk to the dog	**4**	3	5
4	To what extent do you understand what your dog tries to tell you, if	It vocalises	**4**	4	5
		it uses body signs	**4**	4	5
5	To what extent do the following behaviors imply that your dog does not understand the task (your instructions)?	Looks at you	**4.5**	4	5
		performs something else	**4**	3	4
		approaches you	**4**	3	5
		vocalises	**3**	2	4
		displacement behaviors	**3**	2	4
		does nothing	**1**	1	2. 5
6	To what extent do the following behaviors help to realize that your dog is willing to execute the requested task, but for some external reason it cannot do so?	Looks at you	**4.5**	3.5	5
		trying hard, not giving up	**4**	3	5
		approaches you	**4**	3	5
		performs something else	**3.5**	3	4
		displacement behaviors	**3**	2	4
		vocalises	**3**	1.5	4.5
		does nothing	**1**	1	2
7	To what extent do the following behaviors soothe your anger/disappointment, if your dog cannot execute the requested task for some reason?	Looks at you	**4**	3	5
		trying hard, not giving up	**5**	4	5
		approaches you	**4**	3	5
		performs something else	**3.5**	3	4
		displacement behaviors	**2**	1	3
		vocalises	**1.5**	1	3
		does nothing	**1**	1	1
8	To what extent can you imagine having an assistance robot helping you instead of a dog?		**2**	1	3
9	To what extent do you think a modern assistance robot would be able to cover the technical tasks currently executed by your dog?		**3**	3	4
10	To what extent do you think a modern assistance robot could be an emotional partner?		**1**	1	2

#### Data analysis

In addition to a descriptive evaluation of assistance dog–owner interactions, we compared the effect of the less efficient dog behavior in case of insoluble problems (based on Os' scores) with not responding at all (the assumed default state of a robot, which can occur in case of dogs as well) using Wilcoxon signed ranks tests. Spearman's rho correlations were used to investigate the associations of predicted robot competencies with the acceptance rate of assistance robots.

### Result

**Figure 6:**
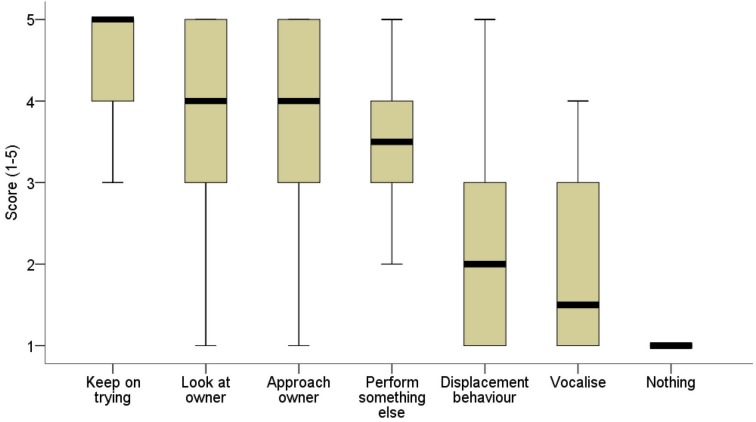
**The distribution of scores owners gave to rate their assistance dog's responses depending on the efficiency of the behavior in attenuating their annoyance/disappointment in situations when the dog cannot/does not perform a task (No 7)**. The heavy black lines mark the median (50th percentile), the boxes mark the lower and upper quartiles, and the whiskers mark the minimum and maximum observed values that are not statistically outlying.

The owners were pretty satisfied with the work of their dogs (No 1) and, in addition, they typically considered the dog as an emotional partner (No 2) (see Table 2). They communicate verbally to the dog not only to give instructions, but also to ask questions and even just to talk to them (No 3). They reported understanding both the vocalizations and body language of the dog to a fairly great extent (No 4). When dogs do not understand what to do or cannot perform the task, owners consider several dog behaviors useful to solve the situation (No 5, 6 – see Table 2; No 7 – see Figure 6). Looking at and approaching the owner in problem situations seem to be basic forms of communication initiation. Trying to perform a different task (offering something else than requested) also proved to be a good strategy. Even displacement behaviors (yawning, stretching, licking mouth, etc.) and different types of vocalizations seem to help O significantly more in comprehending the situation than simply not performing the task (No 5—displacement vs. nothing: *Z* = −4.674, *p* < 0.01; No 6—vocalization vs. nothing: *Z* = −3.812, *p* < 0.001).

Assistance dogs can attenuate Os' disappointment or anger in problem situations efficiently by trying hard to perform even an insoluble task, looking at/approaching O, or by performing some other task. It seems that responding with displacement behaviors or even vocalizing is more effective than doing nothing (vocalizing vs. nothing: *Z* = −3,333, *p* < 0.001). (No 7; see Figure 6)

**Figure 7:**
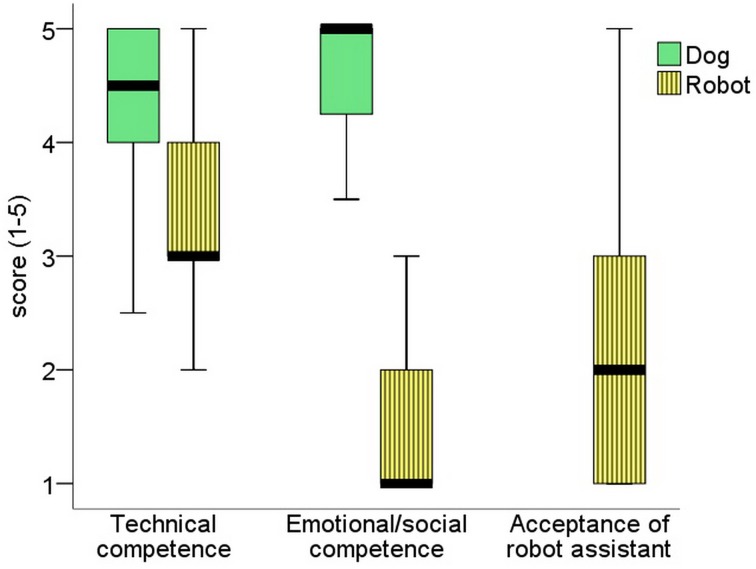
**The first two pairs of boxes present the scores owners gave to evaluate their dog's work and emotional support (questions No 1, 2) and predict future assistance robot's technical and social competences (No 9, 10)**. The last box displays owners' attitude to use a modern assistance robot instead of the dog (No 8). The heavy black lines mark the median (50th percentile), the boxes mark the lower and upper quartiles, and the whiskers mark the minimum and maximum observed values that are not statistically outlying.

The questions regarding future assistance robots revealed that Os anticipated a modern robot to be able to cover the technical tasks of an assistant dog to a moderate/high extent (No 9). However, they clearly cannot envision a future assistance robot as an emotional partner (No 10). Both predictions seem to contribute to their scores rating to what extent they can imagine having a robot assistant instead of a dog (No 8) (acceptance x technical competence: *r* = 0.372, *p* = 0.018; acceptance x social competence: *r* = 0.471, *p* = 0.002), but predictions about the technical and social competence have opposite effects (Figure 7).

## Discussion

Although, in addition to human caretakers, nowadays it is mostly assistance dogs that help people with disabilities in their every-day life, current research on social robotics has striven to make a significant breakthrough in this field. We have proposed to investigate the behaviors of assistance dogs to design acceptable robotic helpers based on the evidence that dog–human interactions provide a promising model system to study the emergence of social competence in general (Topál et al., 2009).

In Study 1, observations of owner-assistant dog dyads' cooperation in a fetch and carry task showed that even the crucial elements of the interaction, such as attention getting behaviors and the human partner's verbal communication, can show both context specific and dyad specific features (for similar results, see Faragó et al., 2014). The contribution of simple verbal instructions to the successful execution of different tasks (fetching, carrying, placing) needs further analyses, but importantly, during the interactions, owners' verbal activity was complemented by non-verbal communication. This draws attention to the significance of the ability for parallel perception and processing of human verbal and non-verbal communication, for instance the need to be able to interpret gestures in assistance robots (see e.g., Nandy et al., 2010). We revealed that joint attention was absolutely required for the initiation of the interaction. As a rule, owners draw their dog's attention before giving any instruction, and they look at each other before performing further actions. This implies that even very simple non-humanoid robots should show their “attentional state” by orienting their body and/or “head” to show “attention” toward the human, as this facilitates the initiation of the interaction.

Pointing at an object to be manipulated by the dog also proved to be a typical action, and dogs could successfully rely on this gesture. For dogs, there is typically no special training required; they are successful in the spontaneous use of the human pointing gesture (Miklósi et al., 2003). Dogs' ability to select the requested object from several objects placed close to each other needs further investigation, but we suggest that the interpretation of simple pointing gestures (with a special attention to pointing with the head in case of persons with a mechanical wheelchair) could be a very useful skill for assistance robots, and on-going research (e.g., Nickel and Stiefelhagen, 2007) should proceed forward in this area applying results from dog cognitive studies (e.g., Lakatos et al., 2012).

During the carrying task, the dogs applied different strategies depending on their experiences. Although there was no significant difference between the age of the novice and experienced groups we cannot exclude some effect of age in this respect. Novice dogs mainly followed the owner toward the target area or walked beside them (Young et al., 2011). Experienced dogs could use more subtle information to find out the correct direction using verbal instructions and, especially in the case of wheelchair users, by extrapolating the direction of the initial movement of the owner to predict the location of the target. This tendency in experienced assistance dogs is important to note, because in real life situations, the ability to navigate the assistant (dog or robot) by wheelchair user persons in front of them, thus not being forced to look back regularly, could be especially useful. Although this particular activity (carrying) is limited to a certain extent by the owner's capabilities, wheelchair-bound owners did not apply more verbal instructions for this task. This implies that the number of commands is not simply linked to the duration or complexity of a task, but rather to the quality of the cooperation.

The interaction of experience and wheelchair use played an important role in the success of placing an object in a predefined place. This was more difficult for the novice wheelchair user group, probably because the usual end of the basket carrying task is at a human and some inexperienced dogs were not used to flexibly combining the behavior elements learnt in different contexts on command (that is, placing the basket and leaving it on the ground). In addition to group level differences, we could observe marked within group (individual) differences during the interactions, suggesting that individual learning in the owner's own environment can enhance and refine such abilities. Thus the ability of future assistance robots to adapt to the capacity and preferences of the user could be of great importance.

In Study 2, dogs' behavior responses were observed in two tests that presented two types of conflict situations when dogs faced an insoluble problem while performing a seemingly simple fetch and carry task. In Test 1, there was no object at the location indicated by the naïve owner, and in Test 2, the object was there, but the experimenter inhibited the execution of the required action. We expected that in addition to trying hard to fulfill the task, dogs would express their confusion and negative inner state in some relevant observable behaviors; either by actively communicating to the owner, or by exhibiting displacement activities. We found that gazing at the owner is not only typical at the beginning of an interaction (Study 1), but also when the dog encounters a problem (Study 2, both Tests 1 and 2). In contrast to Study 1 (when dogs did not vocalize), during the inhibited trials, dogs displayed some specific vocalization, that is, they emitted high-pitched sounds that could reflect their inner state in an explicit manner, which needs no training to be interpreted by humans (Molnár et al., 2010). There was also a tendency to display less explicit forms of expressive behaviors by showing displacement activities. The results of our questionnaire study revealed that the calming value of these behaviors—processed by the owners either consciously or unconsciously—can account for the differences in the humans' reactions to the dogs' failures.

One major message of the results of the inhibited trials is that assistance dogs do not give up easily if they are faced with a “seemingly” insoluble task. Interestingly, dogs tended to manipulate/fetch different objects than the basket, mainly when the basket was not physically there. In both contexts, dogs showed a strong commitment to execute the task or at least the behavioral elements discussed above lent a “busy” appearance to them. According to the questionnaire results, this strong commitment to fulfill the task is an attractive characteristic in the eyes of the owners and can attenuate their disappointment. The incorporation of such behaviors (e.g., hesitantly turning/paddling, moving around close to the place of the task to be executed, looking/moving back and forth between the user and the aimed object, emitting some high pitched sound) in the behavioral repertoire of assistance robots would enhance their similarity to a living helper. More importantly, displaying these behaviors in particular situations would elicit more empathy, understanding, and forgiveness toward the robot assistant on the part of the human users. We stress that we do not envisage robot users to be as sympathetic to an ethologically inspired social robot as to a pet, but suggest employing behaviors which potentially can facilitate some feelings and positive approach on the long run in a human partner.

In sum, we propose that future assistance robots should follow relevant natural behavior sequences during fetch and carry interactions, and that the problem solving strategies of assistant dogs could inspire the development of the relevant functions and social behaviors of assistance robots. We confirmed that it would make human robot interaction more fluent and less stressful if assistant robots could communicate their inability to solve a problem displaying simple behaviors like non-verbal vocalization, gaze alternation, and/or displacement activities rather than simply not performing the task (or giving an error message). Direct questioning of the target population of end-users suggests that the inclusion of assistance dogs' relevant expressive behaviors in the design of future assistance robots could be critical to the success of this emerging assistive technology by decreasing the present negative approach of potential users. Finally, we do not suggest applying the dog-human interactions as a model in all types of social robots or in all possible contexts. Naturally, human-human interactions can be more adequate in many cases. However, we think that the long experience with dogs assisting humans in various situations could give a range of insights for shaping collaboration between agents with heterogeneous capabilities such as humans and robots.

## Author contributions

Márta Gácsi designed the study, Szakadát and Márta Gácsi performed the experiments and analysed the data, Márta Gácsi, Sára Szakadát and Ádám Miklósi wrote the paper.

### Conflict of interest statement

The authors declare that the research was conducted in the absence of any commercial or financial relationships that could be construed as a potential conflict of interest.
